# Quantification of ADHD medication in biological fluids of pregnant and breastfeeding women with liquid chromatography: a comprehensive review

**DOI:** 10.3389/fpubh.2024.1437328

**Published:** 2024-08-07

**Authors:** Lena De Hondt, Charlotte Cosemans, Michelle Plusquin, Debby Mangelings, Ann Van Eeckhaut, Eline Tommelein

**Affiliations:** ^1^Research Group Experimental Pharmacology, Department of Pharmaceutical Chemistry, Drug Analysis and Drug Information, Center for Neurosciences, Vrije Universiteit Brussel, Jette, Belgium; ^2^Centre for Environmental Sciences, Hasselt University, Diepenbeek, Belgium; ^3^Department of Analytical Chemistry, Applied Chemometrics and Molecular Modelling, Vrije Universiteit Brussel, Jette, Belgium

**Keywords:** ADHD, biological liquids, breast milk, liquid chromatography, quantification, dexamphetamine, methylphenidate, atomoxetine

## Abstract

Attention Deficit/Hyperactivity Disorder (ADHD) is a neurodevelopmental disorder that has long been considered a concern only in the pediatric population. However, symptoms often sustain into adulthood and may require medication. For women with ADHD, this also means dealing with the disorder during the reproductive period. Medication safety during pregnancy and breastfeeding is a critical concern, and the potential transfer of ADHD medication to infants remains a topic of scientific interest. The quantification of ADHD medications in both maternal blood and breast milk are vital for understanding their pharmacokinetics and potential exposure risks for (nursing) infants. This review aims (1) to compile and critically assess existing research on the transfer of ADHD medications into breast milk and the potential implications for nursing infants and (2) to provide a comprehensive overview and discussion of the literature regarding the quantification of methylphenidate, amphetamine, atomoxetine, viloxazine, guanfacine, clonidine and bupropion in the blood, urine, oral fluid, and breast milk with liquid chromatography. A literature search was conducted using PubMed, Scopus, and Web of Science, to identify relevant articles published from January 2014 up to December 2023. We illustrate the lack of methods to simultaneously monitor multiple ADHD medications as well as the lack of developed methods for breast milk. Finally, we highlight the need for continued research to refine our understanding of medication transfer into breast milk and potential risks, and to develop clinical guidelines to support mothers with ADHD in making informed choices regarding medication use during pregnancy and lactation.

## 1 Introduction

Attention Deficit/Hyperactivity Disorder (ADHD), a neurodevelopmental disorder characterized by inattention, hyperactivity and/or impulsivity (1), has long been primarily considered a concern only in the pediatric population (2, 3). However, in about 50% of children with ADHD, symptoms persist into adulthood (4), exhibiting a global prevalence of adult ADHD of 2.5–2.8% (5). These adults might at some point require treatment, including medication. In 2023, the first longitudinal multinational study on ADHD medication consumption was published, with an overall increase in use of 9.7% per year from 2015 to 2019, mostly in high-income countries (6). Methylphenidate (MPH), (dex)amphetamine [(d)AMP] and its prodrug lisdexamphetamine (LDX) were consumed the most (6) and are also considered the first line drugs (3). Other options consumed less (6) are atomoxetine (ATX), a selective norepinephrine reuptake inhibitor (3) or a new extended-release form of viloxazine, another norepinephrine reuptake inhibitor (7). Guanfacine (GUA) and clonidine (CLO), which are selective alpha-A2 adrenergic receptor agonists, are also frequently used, as well as bupropion (BUP), a norepinephrine/dopamine-reuptake inhibitor (3).

A subgroup within the adult ADHD population is pregnant and breastfeeding women. In 2022, around 3.88 million (8) and 3.67 million (9) children were born in Europe and the United States, respectively. Using a conservative prevalence of 2.5% (5), this translates into about 97,000 mothers in Europe and 91,750 mothers in the United States with ADHD. In recent years, the treatment of this subgroup has become more prevalent (10–14), raising questions about potential risks for both mother and child. Interestingly, ADHD without pharmacotherapy has been linked to various adverse pregnancy outcomes (15), as the combination of (untreated) ADHD symptoms with the physical and psychosocial effects of pregnancy worsens mental health, academic challenges, and socioeconomic difficulties (16). In general, reviews regarding the safety of ADHD medication during pregnancy conclude that physicians should carefully weigh the risks of medication exposure against the risks associated with untreated ADHD (17–20). Despite some research interest in the use of ADHD medication during pregnancy, research regarding the breastfeeding period is lacking (18, 20–26). In this stage there seems to be a reluctance by both clinicians and women to support and maintain breastfeeding when the mother is taking ADHD medication (27), notwithstanding it being associated with benefits for the mother and her child (28). This is mainly due to concerns about the transfer of the medication into the milk and possible adverse health effects on the infant (27). This leads to temporary, though potentially unnecessary interruption of treatment (2) and reluctance to resume medication post-childbirth (29). Indeed, only about 35% of women restarts their ADHD medication in the 6 months postpartum (12). Data regarding breast milk levels of ADHD medication are therefore limited, as presented in Table 1. However, discontinuing ADHD medication may render risks (12), as ADHD comes with specific challenges toward breastfeeding, such as sensory overload and distractibility. This increases the likelihood of early breastfeeding cessation and missed follow-up healthcare appointments for both mother and child (16, 30).

**Table 1:** Drug levels and clinical outcomes of infants after being breastfed by a mother taking ADHD medication.

Molecule	Findings
MPH	A woman exhibited a BM concentration of 7.9 ng/mL at 4 weeks postpartum taking a 36 mg extended-release tablet per day, leading to an AID of 0.0012 mg/kg/day and a RID of 0.2% (31).
	The mean (95% CI) BM concentration of 3 mothers taking 35–80 mg per day was 19 (9.2) μg/L. The calculated AID was 2.9 (1.4) μg/kg/day, and the RID 0.7 (0.6) % (32).
	Maternal serum concentrations in five samples of one breastfeeding woman taking a 5 mg tablet in the morning and a 10 mg tablet in the afternoon chronically (both immediate-release) were < 0.3, 2.3, 3.8, 1.7, and < 0.3 ng/mL, with higher or similar corresponding BM concentrations of <0.3, 2.4, 5.9, 1.4, and < 0.3 ng/mL. The samples were taken just before taking the dose at noon, and 4, 8, and 21 h after the dose at noon. The mean RID was 0.16% (no AID calculated) (33).
dAMP	The mean (95% CI) BM concentration of five mothers taking 15–40 mg was 244 (181) μg/L. The calculated AID was 37 (27) μg/kg/day, and the RID 9.5 (5.7) % (32).
	The median (IQR) BM levels of four women taking 5 mg daily ranged was 140 (66–313) μg/L with an AID of 21 μg/kg/day and an RID of 5.7% (34).
ATX	The manufacturer received a report of two infants sleeping longer than usual after being breastfed by mothers taking ATX. Drug levels, dosages, duration of maternal therapy, nor infant age were provided (27).
VLX	No information available.
GUA	No information available.
CLO	BM concentrations of five women taking 0.300–0.450 mg/day were found to be 0.8 to 2.8 ng/mL with maternal plasma concentrations between 0.4 to 1.5 ng/mL. No RID nor AID were provided (35).
	BM concentrations were about twice, and infant serum concentrations about half that of maternal serum (dose, serum and milk concentrations, AID and RID not provided). They reported two neonates with hypoglycaemia, one with asymptomatic hypotension, two with apathy syndrome, one with transient feeding problems and one with hyperexcitability, but this was not statistically different from neonates who were not exposed. It was also not clear whether these symptoms were a result of exposure during pregnancy or through breast milk (36).
	A case was reported of an infant, breastfed by a mother taking a daily dose of 0.15 mg CLO for hypertension, presenting a consciousness deficit with drowsiness, hypotonia, and suspected generalized seizures 2 days postpartum and progressive central apnoea from the 5th day postpartum. After cessation of breastfeeding, regression of the symptoms was obtained. No AID nor a RID was provided (37).
BUP	The BM concentrations of four women taking 150–300 mg/day, of which two women were measured twice, ranged from undetectable (< 10 ng/mL) to 120 ng/mL with a mean of 64.1 ng/mL and a high SD of 46.8 ng/mL at peak. Only two samples had detectable concentrations at through of 9.5 and 11.5 ng/mL resulting in an average RID (SD) of 5.7% (3.7%) and it was estimated that the mean (SD) dose the infant received was 21.5 mg (13.9 mg) (38).
	Ten women taking 150 mg and after three days increasing the dose to 300 mg exhibited BM concentrations of 4.2–168.3 ng/mL (mean (SD) of 45.2 ng/mL (49.5 ng/mL)) of BUP, 9.0–242.1 ng/mL (mean (SD) of 104.6 ng/mL (62.2 ng/mL)) of hydroxyBUP, 25.4–142.9 ng/mL (mean (SD) 72.1 ng/mL (38.3 ng/mL)) of erythrohydroBUP and 192.7–1052.1 ng/mL (mean (SD) of 459.0 ng/mL (278.4 ng/mL)) of threohydroBUP seven days after initiation. The average AID and RID were 6.75 μg/kg/day and 0.13% for BUP, 15.75 μg/kg/day and 0.30% for hydroxyBUP, 10.8 μg/kg/day and 0.21% for erythrohydroBUP, and 68.85 μg/kg/day and 1.37% for threohydroBUP, respectively. The total RID was 2.01% (39).
	A case was reported with a mother taking 100 mg three times per day with higher peak BM concentration after one dose of 100 mg (0.189 μg/mL) than in maternal plasma (0.044 μg/L) and in infant plasma (undetectable), concluding that BUP accumulates in the milk. No AID nor RID were reported (40).
	An infant seizure after BUP exposure was reported in a child breastfed by a mother taking 150 mg/day (sustained release). No milk nor plasma concentrations were reported (41).

AID, absolute infant dose; ATX, atomoxetine; BM, breast milk; BUP, bupropion; CI, confidence interval; CLO, clonidine; dAMP, dexamphetamine; GUA, guanfacine; IQR, interquartile range; MPH, methylphenidate; RID, relative infant dose; SD, standard deviation; VLX, viloxazine.

To acquire information about the pharmacokinetics of ADHD medication throughout pregnancy and lactation, monitoring the concentration levels of the mother and assessing the correlation with infant blood levels is required. Therefore, the current review aims to provide a comprehensive overview of the recent literature (published between January 2014 and December 2023) from PubMed, Scopus, and Web of Science regarding the quantification of the most frequently used ADHD medications (i.e., MPH, (d)AMP, LDX, ATX, VLX, GUA, CLO and BUP) and their metabolites with liquid chromatography in biological human fluids, namely blood, plasma, serum, urine, and oral fluid, and we put a specific emphasis on breast milk. Only methods with the aim to quantify these medications in human matrices for ADHD treatment were included to assess their applicability for therapeutic drug monitoring (TDM) of ADHD medication. The results are summarized in Table 2 and the most important findings per (group of) molecule(s) are described here below.

**Table 2:** Studies retrieved with quantitative liquid chromatographic methods for all included medication.

Molecule	Matrix, sample volume	Sample preparation	Internal standard	Instrument	Stationary phase	Mobile phase, elution mode	LLOQ (ng/mL)	Concentration range (ng/mL)	Ref.
MPH	UR, 10 mL	SBME	NA	LC-UV	C8 (250 mm × 4.6 mm, 5 μm)	phosphate buffer (pH 4.6):MeOH:ACN, 56:40:6 (V/V/V), ISO	50	50–5,000	(42)
MPH	PL, 10 mL UR, 10 mL Both 1:3 diluted	three phase HF-LPME	NA	LC-UV	C18 (250 mm × 4.6 mm, 5 μm)	50 mM potassium dihydrogen phosphate in water:MeOH, 57:43 (V/V), ISO	12.0	12.0–5000.0 (both)	(43)
MPH RA	UR, 5 mL	DSPE with PNS	NA	LC-UV	C18 (25 cm × 4.6 mm, 5 μm)	10 mM phosphate buffer (pH 3.5):ACN, 80:20 (V/V) ISO	28.40	30–1,200	(44)
MPH	DBS	PP	MPH-d10	LC–MS/MS, TQD, ESI	C18 (50 mm × 2.1 mm, 3.5 μm)	5 mM ammonium formate in water:0.1% FA in ACN, 80:20 (V/V), ISO	0.200	0.2–25	(45)
MPH EPH RA	BL, 250 μL	SPE	d-threo-MPH-d10 l-threo-MPH-d10 d,l-threo-RA-d10	LC–MS/MS, TQD, ESI	Chiral vancomycin (100 mm × 2.1 mm, 2.7 μm) with precolumn EC-C18 (5 mm × 2.1, 2.7 μm)	0.0125% trifluoroacetic acid (V/V) in deionized water:0.025%:ammonium acetate (W/V) in MeOH, 2:98, ISO	0.5	0.5–500	(46)
MPH RA	OF, 1 mL	D&S	MPH-d9 RA-d10	LC–MS/MS, TQD, ESI	EC-C18 (100 mm × 2.1, 2.7 μm)	0.1% FA and 5 mM ammonium formate in water:0.1% FA in MeOH, GR	0.5 (both)	0.5–75	(48)
(d)AMP	PL, 200 μL	LLE	AMP-d6	LC–MS/MS, TQD, ESI	C18 (150 mm × 2.1 mm, 2.6 μm)	0,05% FA in water:0,05% FA in MeOH, 50:50 (V/V), ISO	2.5	2.5–250	(49)
AMP	UR, 200 μL	MIPs MISPE	AMP-d10	LC–MS/MS, TQD, ESI	C18 (100 mm × 2.1 mm, 3.5 μm)	0.1 M ammonium formate in water:0.1% FA in MeOH, GR	5	5–5,000	(50)
LDX, AMP	OF, 200 μL PL, 100 μL UR, 400 μL	PP	AMP-d5	LC–MS/MS, TQD, ESI	C18 (100 mm × 2.1 mm, 3 μm), with precolumn C18 (4 × 3 mm)	5 mM ammonium formate buffer and 0.1% FA in water:5 mM ammonium formate buffer and 0.1% FA in MeOH, GR	1 (OF, PL) 4 (UR)	1–128 (OF, PL); 4–256 (UR)	(51)
AMP LDX	PL (from 5 mL BL)	PP	AMP-d8 LDX-d4	LC–MS/MS, TQD, ESI	Amide (15 cm × 2.1 mm, 2.7 μm)	10 mM ammonium acetate with FA (concentration not reported) in water:ACN, GR	assessed but NR	dAMP: 2.000–200.000 LDX: 1.000–100.000	(52)
AMP	UR, 75 μL	D&S	AMP-d5	LC–MS/MS, TQD, ESI	Column switching: C18 (110 Å, 10 mm × 2 mm, 5 μm), Lux AMP (150 × 3.0 mm, 3 μm)	0.1 M ammonia (pH 11) in water:ACN, 75:25 (V/V), ISO	0.05	50–25,000	(54)
LDX	BL, 0.2 g UR, 100 μL	PP	AMP-d8	LC–MS/MS, TQD, ESI	C18 (50 mm × 2.1 mm, 1.7 μm)	0.1% ammonia in water:MeOH, GR	NR	0.01–3.0 μg/g (BL) 0.1–100 μg/mL (UR)	(55) achiral method
AMP	UR*, 100 μL	D&S	AMP-d8	LC–MS/MS, TQD, ESI	Chiral vancomycin (250 mm × 2.1 mm, 5 μm)	0.1% acetic acid and 0.02% ammonia in water:0.1% acetic acid and 0.02% ammonia in MeOH:, 5:95 (V/V), ISO	NR	100–25,000	(55) chiral method
AMP	OF, 20 μL	PP	AMP-d5	LC–MS/MS, TQD, ESI	HSS C18 (150 mm × 2.1 mm, 1.7 μm)	20 mM ammonium formate and 0.1% FA (pH 3) in water: 0.1% FA in MeOH, GR	0.9	0.5–500	(56)
LDX	OF, 100 μL	PP	LDX-d4	LC–MS/MS, TQD, ESI	HSS C18 (150 mm × 2.1 mm, 1.7 μm)	20 mM ammonium formate and 0.1% FA (pH 3) in water: 0.1% FA in MeOH, GR	0.0072	0.005–15	(56)
AMP	PL, 250 μL	LLE Deriv.^¥^	AMP-d5	LC-HRMS and LC-HRMS, QD	C18 (100 mm × 2.1 mm, 1.9 μm)	0.012% FA and 5 mM ammonium acetate in water: 0.012% FA and 5 mM ammonium acetate in MeOH, GR	0.024	lAMP: 0.024–25 dAMP: 0.098–100	(57) achiral method
AMP	PL, 250 μL	Deriv. ^¥^	AMP-d5	LC-HRMS, QD	Chiral ß-cyclodextrin (25 cm × 4 mm, 5 μm)	0.012% FA and 5 mM ammonium acetate in water: 0.012% FA and 5 mM ammonium acetate in MeOH, GR	NR	lAMP: 0.024–25 dAMP: 0.098–100	(57) Chiral method
LDX 4OH-AMP	UR, 100 μL	D&S	LDX-d4	LC–MS/MS, QD-Orb	C8 (100 mm × 3, 4 μm)	0.1% FA in water:ACN, GR	LDX: 0.15 4OHAMP: 5	LDX: 0.15–11 4OH: 5–200	(53)
ATX	SER, 100 μL	SPE	D-clomipramine	LC-UV	Column switching: cyanopropyl 20 μm extraction column, C8 (12.5 cm × 4 mm, 5 μm)	40 mM disodium hydrogen phosphate in water (pH 3.35):ACN, 64:31 (V/V), ISO	5	5–2000	(58)
ATX	PL, 500 μL	Double LLE	Mianserine	LC-UV	C8 (150 × 2.1 mm, 3.5 μm) with precolumn (12.5 × 2.1 mm, 5 μm)	30 mM potassium dihydrogen phosphate in water (pH 5.1):ACN, 66:34 (V/V), GR	2.0	2.0–1,440	(59)
ATX	PL, 50 μL	PP	ATX-d3	LC–MS/MS, TQD, ESI	C18 (50 mm × 2.1 mm, 2.6 mm) with precolumn C18 (4 × 2.0 mm)	5 mM ammonium acetate and 0.1 mM FA in water:5 mM ammonium acetate and 0.1 mM FA in MeOH, GR	0.5	0.500–2000	(60)
ATX	BM, 100 μL	PP	ATX-d3	LC–MS/MS, TQD, ESI	C8 (30 mm × 3 mm, 2.6 μm) with precolumn (50 mm × 2.1 mm, 1.7 μm)	0.1% of FA in water (pH 2.38): 0.1% of FA in ACN, GR	0.5	0.5–500	(61)
GUA	PL, 100 μL	PP	GUA-^13^C-^15^N_3_	LC–MS/MS, TQD, ESI	C18-PFP (75 mm × 4.6 mm, 3 μm)	0.1% FA and 10 mM ammonium formate in water: ACN, GR	0.05	0.0500–10.0	(62)
CLO	PL, 1 mL	LLE	Donepezil	LC–MS/MS, TQD, ESI	C18 (2.1 mm × 50 mm, 3 μm)	0.1% FA in water: 0.1% FA in MeOH, 40:60 (V/V), ISO	0.02	0.02–6.00	(63)
CLO	PL, 0.1 mL	PP	/	LC–MS/MS, TQD, ESI	C18 (2.1 mm × 30 mm, 3.5 μm)	0.2% FA in water:0.2% FA in ACN: 40:460 (V/V), ISO	0.010	0.01–10.0	(64)
MPH dAMP ATO	SER OF Both 1 mL (MPH, dAMP), 100 μL (ATX)	LLE	/	LC-FL	C18 (150 mm × 4.6 mm, 3 μm) with precolumn C18 (4 × 2.0 mm)	Water:ACN, GR	MPH: 2.5 (SER), 5.0 (OF) dAMP: 1.3 (both) ATX: 31 (SER), 12 (OF)	MPH, dAMP: 2.5–80 (SER) 5–160 (OF) ATO: 62.5–2000 (SER) 93.75–3,000 (OF)	(65)
MPH LDX AMP EPH	OF, 250 μL	SPE	MPH-d10 AMP-d11	LC–MS/MS, TQD, ESI	EC-C18 (100 mm × 2.1 mm, 2.7 μm)	5 mM of ammonium formate and 0.01% FA in water:0.1% FA in ACN, GR	MPH, AMP, EPH: 0.5 LDX: 5	MPH, EPH, AMP: 0.5–100 LDX: 5–500	(66)
AMP MPH RA 4OH-AMP	UR, 100 μL	D&S	AMP-d8 RA-d5	LC–MS/MS, TQD, ESI	C18 (150 mm × 2.0 mm, 5 μm)	0.2% FA in water:ACN, GR	MPH: 2 AMP, RA: 40 4OH-AMP: 20	4OH-AMP: 20–1,500 AMP, RA: 40–3,000 MPH: 2–150	(67)

ACN, acetonitrile; DBS, dried blood spots; DSPE, dispersive solid phase extraction; D&S, dilute-and-shoot approach; EC, endcapped; EPH, ethylphenidate; ESI, electrospray ionization; FA, formic acid; GR, gradient; HRMS, high-resolution mass spectrometry; HSS, high strength silica; IS, internal standard; ISO, isocratic; LLE, liquid–liquid extraction; LLOQ, lower limit of quantification; MeOH, methanol; MISPE, micro-solid phase extraction; MP, mobile phase; NA, not applicable; NR, not reported; PFP, pentafluorophenyl; PNS, polymeric nanospheres; PP, protein precipitation; QD, quadrupole; QD-Orb, quadrupole-orbitrap; RA, ritalinic acid; SBME, solvent bar microextraction; SP, solid phase; SPE, solid phase extraction; TQD, triple quadrupole; 4OH-AMP, 4-hydroxyamphetamine. *A chiral method for BL was also developed but with GC–MS/MS, which is not within the scope of this review. ^¥^ Derivatization with (S)-N-(heptafluorobutyryl)-prolyl chloride.

## 2 Liquid chromatographic methods for the quantification of ADHD medication in biological samples

### 2.1 Methylphenidate

Six studies that report on a quantitative assay for MPH were retrieved (Table 2), of which three papers used a LC-UV method for the quantification of MPH in urine. All three focused on the development of alternative sample preparation methods, i.e., solvent bar microextraction (SBME) (42), three phase hollow fiber liquid phase microextraction (HF-LPME) (43), and dispersive solid-phase extraction (DSPE) (44). The latter method also allowed the quantification of the inactive metabolite ritalinic acid (RA), which is advantageous for drug screening or patient compliance purposes as MPH has a short half-life (~2.5 h). All three methods required a large sample volume (3–10 mL) and the obtained detection limits were relatively high for sensitive analysis in low volume biological samples.

Two LC–MS/MS methods were developed for MPH quantification in blood. Ghandi et al. (45) developed a method for analyses of dried blood spots (DBS) in pharmacokinetic studies, although the method was not tested on real samples. This approach would be interesting for testing infant blood levels as it only requires low volumes (5 or 10 μL). They used a simple protein precipitation (PP) step. Smith et al. (46) applied mixed-mode solid-phase extraction (SPE) followed by chiral LC–MS/MS for quantification of the enantiomers of MPH and its metabolites ethylphenidate (EPH) and RA in blood samples. EPH is formed by transesterification with alcohol when administered concomitantly (47). A chiral stationary phase with vancomycin as chiral selector was used, thereby excluding the necessity of derivatization reagents to separate the enantiomers. The deuterated internal standards were able to compensate for the significant matrix effects observed (46).

Mulet et al. (48) developed a dilute-and-shoot LC–MS/MS method for MPH and RA quantification in oral fluid. The quick method facilitates non-invasive sampling and monitoring of MPH levels in patients with ADHD. Using stable isotope labeled internal standards (SIL-ISs) was recommended because ion suppression was observed. The authors also recommend storing the oral fluid samples at −20°C and analyzing them as soon as possible, particularly in the case of low sample concentrations (48).

### 2.2 (lis)(dex)amphetamine

Few methods are published for the quantification of AMP or LDX levels in light of ADHD treatment (Table 2). Most often, they are analyzed together with a large panel of other analytes in the context of drug abuse. These methods have not been included in this review as ranges differ significantly. Herbrink et al. (49) developed a LC–MS/MS method to quantify dAMP in plasma using double liquid–liquid extraction (LLE) (plasma-organic and organic-water) combined with snap-freezing. The applicability of the method was shown in clinical studies. El-Beqqali et al. (50) developed a molecularly imprinted polymer-sol–gel (MIPs) deposited on a tablet of polyethylene substrate for micro-solid phase extraction and LC–MS/MS analysis of AMP in urine samples. Each tablet could be used at least 20 times without changes in extraction efficiency.

Two LC–MS/MS methods were described for the simultaneous quantification of LDX and its metabolite AMP. The first method by Comiran et al. (51) analyzed both compounds in oral fluid, plasma and urine after a simple sample preparation step, comprising dilution, PP and filtration. Interestingly, LDX was detected in all three matrices 2 h after administration of 70 mg LDX. The use of matrix-matched calibration curves was important to minimize the impact of the matrix effect, but the method would further benefit from adding a SIL-IS for LDX in oral fluid, as the used IS AMP-d5 did not compensate. The second method by Rizea-Savu et al. (52) used PP as sample preparation to assess both levels of LDX and AMP in plasma in a bioequivalence study. Absolute numbers for LDX plasma concentrations were not provided but were in accordance (1 h after administration of LDX) with the study of Comiran et al. (51), although the dose was not provided.

It is well-documented that many ADHD patients struggle with addiction to drugs of abuse. Thevis et al. (53) proposed a sensitive LC–MS/MS method able to quantify low LDX levels (LLOQ: 0.05 ng/mL) in urine to distinguish LDX use from AMP abuse in doping control, as approximately 2% of the prodrug is eliminated intact into urine. LDX could be quantified up to 6 h and detected up to 11 h after administering a low therapeutic dose. Four papers (54–57) describe the use of chiral LC–MS/MS analysis of clinical samples to monitor ADHD medication compliance and abuse of AMP concomitantly (Table 2). In AMP treatment, single dAMP is often administered, as it has stronger stimulant properties than lAMP, which presents more cardiovascular and peripheral effects (54). By quantifying both enantiomers, the use of AMP for ADHD could therefore be distinguished from AMP abuse. Hädener et al. (54) described a column-switching LC–MS/MS method for AMP in urine, combining online sample purification on a C18 trapping column and chiral separation on a polysaccharide-based chiral column. Chermá et al. (55) showed that LDX only converts into dAMP and not in lAMP in the blood circulation. Only dAMP concentrations should therefore be detected in patients taking LDX. A similar approach was used by Böttcher et al. (56). First, an achiral analysis of AMP and LDX in oral fluid was performed. Secondly, illicit drug use was assessed using a qualitative chiral method for AMP. Instead of analyzing on a chiral stationary phase, Leis et al. (57) derivatized AMP with (S)-N-(heptafluorobutyryl)-prolyl chloride to form diastereomers that could be separated on an achiral stationary phase. They compared their newly developed LC-high resolution MS method for the enantioselective analysis of AMP in plasma with their previously developed GC method (57) and obtained comparable results.

### 2.3 Atomoxetine

Four quantification methods for determining ATX in biological samples were published (Table 2). Two LC-UV methods were described for quantifying ATX in serum (58) and plasma (59), with LLOQs of 5 and 2 ng/mL, respectively. The method of Ruppert et al. (58) included PP as sample pretreatment step, while a double LLE was performed in (59) leading to a preconcentration of approximately 3 times. Xia and Guo (60) developed a LC–MS/MS method to quantify ATX in 50 μL plasma samples using PP as sample pretreatment. LC–MS/MS for simultaneous analysis of 19 drugs and metabolites in 100 μL breast milk, among which ATX, was described by Monfort et al. (61). PP was used as sample pretreatment as this is more generic compared to SPE. However, the addition of SIL-ISs was necessary to correct for matrix effects. The method is intended to be used in studies to obtain knowledge on drug transfer into breast milk, but results on ATX have not yet been published.

### 2.4 Viloxazine

No studies describing assays in human matrices were retrieved.

### 2.5 Guanfacine

Only one study was retrieved, reporting a LC–MS/MS able to quantify GUA in plasma (100 μL) for a bioequivalence study of extended release tablets for ADHD (62) (Table 2). A simple PP was used to purify the samples and determine GUA quantitatively within a range of 0.05–10 ng/mL with sufficient recovery and no significant influence of matrix effects due to the inclusion of GUA-^13^C-^15^N_3_ as an internal standard.

### 2.6 Clonidine

Two papers regarding the quantification of CLO in plasma with LC–MS/MS were found (Table 2). In the first paper (63), plasma was extracted with diethylether, evaporated and reconstituted in ACN with formic acid. The matrix effects for CLO were negligible, but ion suppression was observed for the IS donepezil. This confirms that using SIL-ISs is the method of choice to efficiently correct for matrix effects. The second paper (64) used PP as sample pretreatment for analysis in plasma. Although no IS was included, the validation characteristics seem to comply. However, the paper lacked sufficient information. Both methods were used in bioequivalence studies for transdermal patches (63) and tablets (64).

### 2.7 Bupropion

No methods for the quantification of BUP regarding ADHD treatment have been published.

### 2.8 Multidrug assays

In this section, methods are described that were developed for the analysis of multiple ADHD medications at a time are described (Table 2). Stegmann et al. (65) developed a method to simultaneously quantify MPH, dAMP and ATX in serum and oral fluid. They used LC with fluorescence detection (FL) as it is more cost-effective than LC–MS/MS and as many routine quantification methods still use a HPLC system with a UV or FL detector. However, after LLE of the samples an additional derivatization with 4-(4,5-diphenyl-1H-imidazol-2-yl) benzoyl chloride (DIB-Cl) was needed for fluorescent labeling. No clear correlation between serum and oral fluid could be found for MPH (*n* = 13) and dAMP (*n* = 4), even though it was detected in all patients. In addition, ATX (*n* = 1) was not detected in oral fluid (LOD 5.9 ng/mL). However, the sample size (*n* = 18) was relatively small, and no final conclusions could be made about the correlation between serum and oral fluid. Later, Smith et al. (66) reported an LC–MS/MS method for oral fluid, capable of quantifying AMP, LDX, MPH and its transesterification metabolite ETH. They used SPE combining hydrophobic and cation exchange extraction. Matrix effects were significant, but the different SIL-ISs were able to adequately correct for this. The correlation between oral fluid and blood concentrations was not assessed. A LC–MS/MS method for the simultaneous quantification of MPH and its metabolite ritalinic acid (RA), and AMP and its metabolite 4-hydroxyamphetamine (4-OHAMP) in urine was developed and validated by Kwon et al. (67). The urine samples were centrifuged, diluted with water, the IS was added and the sample was then injected. The applicability of the method was shown for detecting ADHD medication abuse instead of treatment improvement.

## 3 Discussion

In general, recent methods to quantify ADHD medication with liquid chromatography in biological matrices are limited. Most papers retrieved during this literature review are focused on forensic analysis, drug abuse, or other treatments with doses that are unrepresentative for ADHD patients. The included papers mostly reported LC–MS/MS methods, emphasizing their importance in biological sample analysis. In five cases, LC-UV was used as an analysis technique (42–44, 58, 59), but LLOQ values were always higher than for LC–MS/MS methods. LC coupled to fluorescence detection was used once for the simultaneous determination of MPH, dAMP and ATX (65). Although increased sensitivity was obtained, an additional sample preparation step, i.e., derivatization, was required next to LLE to obtain fluorescent molecules. Therefore, LC–MS/MS remains the method of choice for developing a sensitive and selective method for monitoring ADHD medication transfer to breast milk.

During the development of LC–MS/MS methods, the presence of possible matrix effects should be carefully considered. As observed in most studies, these matrix effects are adequately corrected when using matrix-matched calibration curves and the addition of SIL-ISs. Only two LC–MS/MS methods (63, 64) did not include a SIL-IS. Only once a ^13^C-^15^N IS was selected (62), in all other methods deuterated forms were used as SIL-ISs, probably because of their easier availability and lower cost.

Depending on the sample type, different sample pretreatment options are available and/or needed. For urine and oral fluid samples, a dilute-and-shoot approach is possible, but for plasma samples PP is minimally needed (51, 52, 60, 62, 64), especially in LC–MS/MS methods. PP is frequently used due to its simplicity, and the mass spectrometer adds an additional level of selectivity. However, sometimes additional sample preparation is needed (49, 57, 59, 63). Classically, LLE or SPE are used, both having their advantages and disadvantages. Interestingly, novel micro-extraction techniques including SBME (42), HF-LPME (43), and DSPE (44) have been investigated. Combining them with more sensitive LC–MS/MS methods would be interesting to further evaluate their potential for volume-limited samples. While high selectivity is offered by MIPs, this may limit the number of compounds that can be used for extraction, making them less ideal for TDM (50). The single paper on breast milk used PP (61), while SPE seems the preferred method to efficiently remove lipids, proteins and salts and reduce matrix effects. Moreover, the addition of SIL-ISs compensates for matrix effects.

Almost all achiral methods used a C18 column, although one paper used an amide column (52). Some studies used C8 columns. For example, Monfort and colleagues (61) specifically chose a C8 column as an analytical column to avoid too many phospholipids in breast milk sticking to the column and deteriorating the chromatographic performance. Typically, conventional or narrowbore columns have been used, indicating that sensitivity can still be improved by using miniaturized systems, which would be beneficial for breast milk analysis, since we expect lower medication levels than in maternal plasma. Enantioselective analysis of AMP can also be important (46, 54–57) to distinguish between use and abuse of ADHD medication, but this is not necessary for determining the concentration in breast milk.

Most published articles reported methods for MPH, AMP and ATX. Only one method for GUA and two for CLO in plasma were found. Other methods for CLO were applied in hypertensive (68–73) or analgesic treatment (74, 75) or on mouse plasma (76). No methods to quantify VLX and BUP for treating ADHD in human matrices were published. However, one study described a chiral LC–MS/MS analysis of VLX in rat plasma (77). After PP, the enantiomers were separated on a cellulose tris-(3,5-dichlorophenylcarbamate) column. Methods retrieved for BUP were published for the treatment of depression or smoking cessation, which is reasonable as BUP is widely used off-label for ADHD (3).

When developing an analysis method for TDM, it preferably enables the quantification of multiple ADHD medications at the same time, as a considerable part of patients uses more than one type of ADHD medication (12). Some methods with multiple ADHD medications were published (65–67), but no method exists that considers all available ADHD medications. An ideal method should be able to detect a wide range of levels, so it is applicable to both patients and their offspring, the latter who are expected to exhibit much lower plasma levels. Such an analytical method is currently lacking. Moreover, a method combining medication for ADHD and comorbidities, such as depression or anxiety (78), requires further research.

To date, only one method in breast milk regarding the treatment of ADHD (i.e., ATX) has been published in the last 10 years. This method is simple and has a high sensitivity, providing a promising first step to increase knowledge regarding the transfer of ADHD medication into breast milk. Also, two LC–MS/MS methods to assess AMP abuse in breast milk were published (79, 80).

As it is not ethical to perform multiple invasive sampling in infants, and because of its simplicity, oral fluid or urine testing might be a non-invasive alternative for TDM (65, 81). This however requires a computational modeling of the correlation between these matrices and plasma, serum or breast milk, about which further research is still necessary. Only one of the included papers (65) investigated a correlation between serum and oral fluid with no clear conclusions. Comiran et al. however reported a study (82) using their developed method (51) and found a statistically significant correlation of 0.87 between plasma and oral fluid for AMP but not LDX. They also observed a high variation of AMP concentrations in urine, which might be due to the lack of pH control. They also observed low recovery of intact LDX.

In conclusion, this review highlights the need for continued research to refine our understanding of medication transfer into breast milk and potential risks, and to develop clinical guidelines to support mothers with ADHD in making informed choices regarding medication use during pregnancy and lactation. To know how much the (unborn) child is exposed and to support the decision to (re)start ADHD medication, clinical practice is still in need of a fast and specific TDM method for different biological matrices. Furthermore, it is unclear whether a correlation exists between maternal blood and fetal blood, between maternal blood and breast milk, and between blood and oral fluid (65, 81). This makes evidence-based decisions regarding ADHD medication use during the reproductive period challenging.

## Author contributions

LH: Conceptualization, Investigation, Methodology, Project administration, Visualization, Writing – original draft, Writing – review & editing. CC: Writing – review & editing. MP: Writing – review & editing. DM: Methodology, Writing – review & editing. AE: Conceptualization, Methodology, Project administration, Supervision, Visualization, Writing – original draft. ET: Conceptualization, Methodology, Project administration, Supervision, Visualization, Writing – review & editing.
